# Glutamatergic dysfunction in Schizophrenia

**DOI:** 10.1038/s41398-022-02253-w

**Published:** 2022-12-03

**Authors:** Andreas O. Kruse, Juan R. Bustillo

**Affiliations:** Department of Psychiatry and Behavioral Sciences, University of New Mexico, Albuquerque, NM 87131 USA

**Keywords:** Schizophrenia, Molecular neuroscience, Predictive markers

## Abstract

The NMDA-R hypofunction model of schizophrenia started with the clinical observation of the precipitation of psychotic symptoms in patients with schizophrenia exposed to PCP or ketamine. Healthy volunteers exposed to acute low doses of ketamine experienced mild psychosis but also negative and cognitive type symptoms reminiscent of the full clinical picture of schizophrenia. In rodents, acute systemic ketamine resulted in a paradoxical increase in extracellular frontal glutamate as well as of dopamine. Similar increase in prefrontal glutamate was documented with acute ketamine in healthy volunteers with ^1^H-MRS. Furthermore, sub-chronic low dose PCP lead to reductions in frontal dendritic tree density in rodents. In post-mortem ultrastructural studies in schizophrenia, a broad reduction in dendritic complexity and somal volume of pyramidal cells has been repeatedly described. This most likely accounts for the broad, subtle progressive cortical thinning described with MRI in- vivo. Additionally, prefrontal reductions in the obligatory GluN_1_ subunit of the NMDA-R has been repeatedly found in post-mortem tissue. The vast ^1^H-MRS literature in schizophrenia has documented trait-like small increases in glutamate concentrations in striatum very early in the illness, before antipsychotic treatment (the same structure where increased pre-synaptic release of dopamine has been reported with PET). The more recent genetic literature has reliably detected very small risk effects for common variants involving several glutamate-related genes. The pharmacological literature has followed two main tracks, directly informed by the NMDA-R hypo model: agonism at the glycine site (as mostly add-on studies targeting negative and cognitive symptoms); and pre-synaptic modulation of glutamatergic release (as single agents for acute psychosis). Unfortunately, both approaches have failed so far. There is little doubt that brain glutamatergic abnormalities are present in schizophrenia and that some of these are related to the etiology of the illness. The genetic literature directly supports a non- specific etiological role for glutamatergic dysfunction. Whether NMDA-R hypofunction as a specific mechanism accounts for any important component of the illness is still not evident. However, a glutamatergic model still has heuristic value to guide future research in schizophrenia. New tools to jointly examine brain glutamatergic, GABA-ergic and dopaminergic systems in-vivo, early in the illness, may lay the ground for a next generation of clinical trials that go beyond dopamine D2 blockade.

## Introduction

Schizophrenia is a psychotic disorder that is characterized by significant loss of function and continues to constitute a significant disease burden globally [1]. The disorder is characterized by positive symptoms such as hallucinations, delusions, disorganized behavior, as well as gradual loss of cognitive functions and negative symptomatology [2]. The pharmacological efforts to treat schizophrenia have for many years largely focused on dopamine 2 receptor antagonism, which constitutes the primary mediatior of antipsychotic effect of efficacious drugs [3, 4]. However, there are shortcomings of current antipsychotics. Notably, the limited effect on cognitive decline and negative symtpoms typically associated with chronic stages of schizophrenia, as well as a sub-population of schizophrenia patients who are largely nonresponders to dopaminergic antipsychotics [5]. Such limited efficacy, as well as the unilateral approach to pharmacological antipsychotic treatment identified the need for an alternate neurochemical target for schizophrenia.

The neurotransmitter glutamate and its target, the N-methyl-D-aspartate receptor (NMDA-R), has a central role in fundamental brain functions including neural plasticity, neural network formation, and central nervous system repair, whereas excessive levels of glutamate are associated with excitotoxicity and neural degeneration [6]. Positive symptoms of schizophrenia has also been replicated by antagonism of the NMDA-R by substances such as phencyclidine (PCP) and ketamine [7]. This phenomenon, combined with observed neurodegenerative changes in animal models of NMDA-R hypo-function, to which atypical antipsychotics showed neuroprotection, gave rise to the NMDA-R hypofunction hypothesis of schizophrenia [8]. Since its initial postulation more than three decades ago, testing of the model has been at the forefront of schizophrenia research.

This paper highlights advances in several research fields relating to glutamatergic function as it pertains to schizophrenia and other psychotic disorders. We first review the basic physiology of glutamatergic neurotransmission and its perturbation in the context of the NMDA-R hypofunction model.

We constrained our review to five general areas of research in which measurement or manipulations of the glutamatergic system in groups of schizophrenia patients have been implemented: neuro-imaging (PET and ^1^H-MRS), CSF and peripheral blood, post-mortem, genetics, and clinical trials. We implemented several literature searches to identify relevant studies using PubMed (search from 1/1/1980 to 9/31/2022) using the following search terms: schizophrenia AND MRS AND glutamate; schizophrenia AND PET AND glutamate; schizophrenia AND post-mortem AND glutamate); schizophrenia AND CSF AND glutamate; schizophrenia AND antibodies AND glutamate OR NMDA; schizophrenia AND clinical trial AND glutamate; schizophrenia AND GWAS; and schizophrenia AND CNV. We also supplemented these with manual searches of the referenced articles. We identified the most recent meta-analyses and summarize these to provide an overview of the magnitude and consistency of findings for each approach. When meta-analyses were not available, we identified the most recent narrative reviews; when these were not available, we focused on the largest studies. Finally, we often chose particularly informative individual studies (because of their sample size, novel technology or longitudinal design) to complement meta-analyses and reviews. Finally, we elaborate on the meaning and significance of this vast literature as well as its implication for future approaches.

## The glutamatergic synapse and the NMDA-R-hypofunction model of schizophrenia

### Physiology

**Fig. 1:**
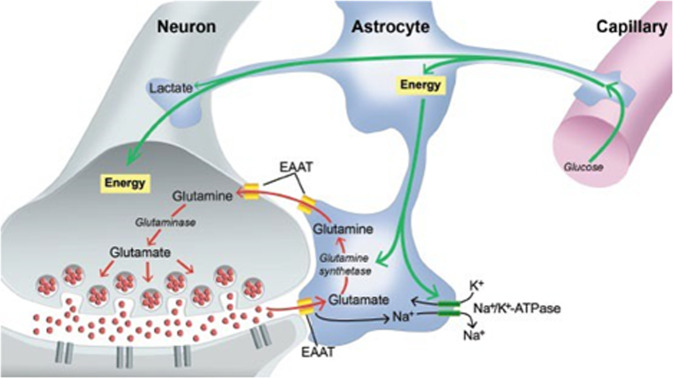
Glutamate/glutamine cycle. Glutamate removed from synaptic cleft by excitatory amino acid transporters (EAATs) into astrocytes and converted into glutamine by enzyme glutamine synthetase. Glutamine is then transported out of astrocytes into nerve terminals and converted back to glutamate via glutaminase (reproduced from Magistretti PJ, Pellerin L, Rothman DL, et al. (1999). Energy on demand. *Science* 283: 496–497 permission from The *American Association for the Advancement of Science* requested).

Glutamate is the major excitatory neurotransmitter in the central nervous system. It is the amino-acid with the highest concentration in the brain (5–15 mmol/kg brain tissue [9]). Hence, most of synapses use glutamate. However, glutamate concentrations have to be kept within a tight physiologic range because high concentrations directly result in excitotoxic neuronal death [10]. Therefore the regulation of cycling of glutamate through the synapse has several redundant mechanisms to optimize signal transmission, economy, and prevention of toxicity (Fig. 1). Glutamate released in the synaptic cleft from presynaptic terminals stimulates post-synaptic neurons. Re-uptake from the cleft by the excitatory amino acid transporters (EAAT) into astrocytes terminates post-synaptic receptor activation.

In the astrocyte, the majority of glutamate is converted to glutamine (Gln) by glutamine synthetase. A smaller proportion is metabolized to alpha-ketoglutarate which enters the Krebs cycle. Glutamine is then transferred back to the presynaptic terminal and converted by glutaminase into glutamate. Glutamate is then re-packaged into pre-synaptic vesicles for further neurotransmission. Hence, the glutamate/glutamine cycling through the synapse is intimately linked to aerobic metabolism and accounts for greater than 80% of cerebral glucose consumption [11].

Glutamate activates G protein-coupled metabotropic receptors (mGluR) and ionotropic receptors. mGluRs modulate cell synaptic transmission via secondary messenger systems and are of three types: group one (mainly post-synaptic, mGluR_1_ and mGluR_5_) and groups two (mGluR_2_ and mGluR_3_) and three (mGluR_4_, mGluR_6_, mGluR_7_, and mGluR_8_) are largely presynaptic and regulate glutamate release [12].

Ionotropic receptors are ligand gated ion channels, account for the majority of excitatory neurotransmission and have a key role in synaptic plasticity. They are divided into three subtypes each with various subunits: NMDA-R (GluN_1_, GluN_2_A, GluN_2_B, GluN_2_C, GluN_2_D, GluN_3_A, and GluN_3_B); α-amino-3-hydroxy-5-methyl-4-isoxazolepropionic acid receptor (AMPA-R; GluA_1_, GluA_2_, GluA_3_, and GluA_4_); and kainite receptors (GluK_1_, GluK_2_, GluK_3_, GluK_4_, and GluK_5_). Each subunit of the metabotropic and ionotropic receptors is encoded by a single gene [13].

NMDA-R have various combinations of subunits compositions. The main NMDA-R includes two GluN_1_ and two GluN_2_ subunits. NMDA-R de-sensitize more slowly than AMPA-R and KA-R. NMDA- R activation requires: initial activation of an AMPA-R to remove magnesium from a GluN_1_; occupancy by glycine or D-serine on a GluN_1_; and binding of glutamate to a GluN_2_ to allow influx of calcium into the post-synaptic terminal. Ketamine and PCP bind on the transmembrane domain of the NMDA-R resulting in hypofunction [14].

### NMDA-R blockade

So-called dissociative anesthetics, which control pain while maintaining consciousness, were also found to induce gross and temporary psychological symptoms (ie: “psychotomimetic” effect), including dissociative symptoms (e.g., sensory distortions, derealization, depersonalization). As PCP and ketamine were used recreationally, primarily for their dissociative properties, they were also observed to induce both positive symptoms (e.g., delusions, hallucination), negative symptoms (social withdrawal and blunting of affect) as well as some of the cognitive deficits present in schizophrenia. A recent meta-analysis showed a transient but significant increase in these symptoms among healthy volunteers (HV) after infusion of racemic ketamine [15].

These findings are consistent with early reports of PCP induced clinical signs mimicking schizophrenia [16–18]. In a small population of antipsychotic naïve patients with schizophrenia, ketamine infusion elicited a brief exacerbation of both positive and negative symptoms, as well as transient reduction of cognitive domains [19]. Another study encompassing both HV and patients with schizophrenia, showed similar findings with exacerbations of positive symptoms after ketamine infusion to similar extents in both groups, albeit from different baselines [20].

Eventually, it was found that blockade of the NMDA glutamate receptor accounted for the anesthetic and psychotomimetic properties of both ketamine and PCP [21]. However, in awake rats exposed to systemic acute ketamine under frontal lobe micro-dialysis, a somewhat paradoxical increase in extracellular Glu (as well as dopamine) was found [22]. The paradoxical increase in cortical Glu with ketamine has been related to a more sensitive NMDA-R blocking effect on GABAergic than glutamatergic neurons [23]. Hence, it was proposed that hypofunction of NMDA-R on GABA-ergic interneurons reduces their inhibitory effect on glutamatergic pyramidal neurons in the cortex. This disinhibition ultimately leads to excessive presynaptic glutamate release, especially in frontal cortical areas [8]. Consistently, acute NMDA-R blockade with low-dose ketamine increases ^1^H-MRS measured prefrontal glutamatergic metabolites in [24, 25].

Hence, the acute low dose pharmacological NMDA-R blocker challenge mimics the broad symptomatology of schizophrenia and results in increased frontal glutamate in both animals and humans. However schizophrenia is a chronic illness, so more persistent NMDA-R blockade has also been examined.

In rats, chronic (1 month) NMDA-R blockade with low dose PCP resulted in cortical hypometabolism [26] and reduced N-acetylaspartate [27] which have been described in schizophrenia. Similar low dose chronic PCP resulted in reduced frontal glutamate and GABA measured ex-vivo with ^1^H-MRS in rodents [28]. Furthermore, a 1 week low dose PCP exposure resulted in marked reductions of medial frontal dendritic spines and increased astroglia process density [29]. These results with chronic NMDA-R blockade are consistent with some of the post-mortem literature in schizophrenia [30]. Human studies of chronic controlled NMDA-R hypofunction are not ethically feasible. However, in subjects with a history of chronic PCP misuse, pre-frontal hypo-activation has been reported [31, 32].

### Neuroimaging studies

In-vivo neurochemical imaging has rapidly evolved and has been systematically applied over the past 30 years. Following the very successful application of positron emission tomography (PET) to dopamine metabolism, high hopes were placed on the development of glutamate receptor binding radionuclides to examine the NMDA-R-hypofunction model of psychosis. Only one small study by Pilowsky et al. [33], reported reduced NMDA-R binding in the left hippocampus in schizophrenia patients. However, the specificity of the radioligand was questioned. Since then, one more study was published using a metabotropic GluR5 radioligand which found no group differences [34]. Still, new tracers are under development [35]. Recently, a PET tracer for the AMPA receptor was developed [36]. However, the remainder of this neuroimaging study section will focus on the application of ^1^H-MRS to examine glutamatergic abnormalities in schizophrenia.

### ^1^H-MRS: technical considerations

^1^H-MRS is a non-invasive imaging technique that allows the measurement of concentrations of some molecules in brain and other tissues; see Lin et al. 2022 for review of technical issues [37]. The technique uses the same hardware as magnetic resonance imaging (MRI) to measure the radio-waves emitted by protons spinning (resonating) in a changing magnetic field. Depending on the chemical micro-environment surrounding each proton, the radio-wave frequencies will vary lawfully, providing a signature for the proton attached to a particular molecule in the tissue.

**Fig. 2:**
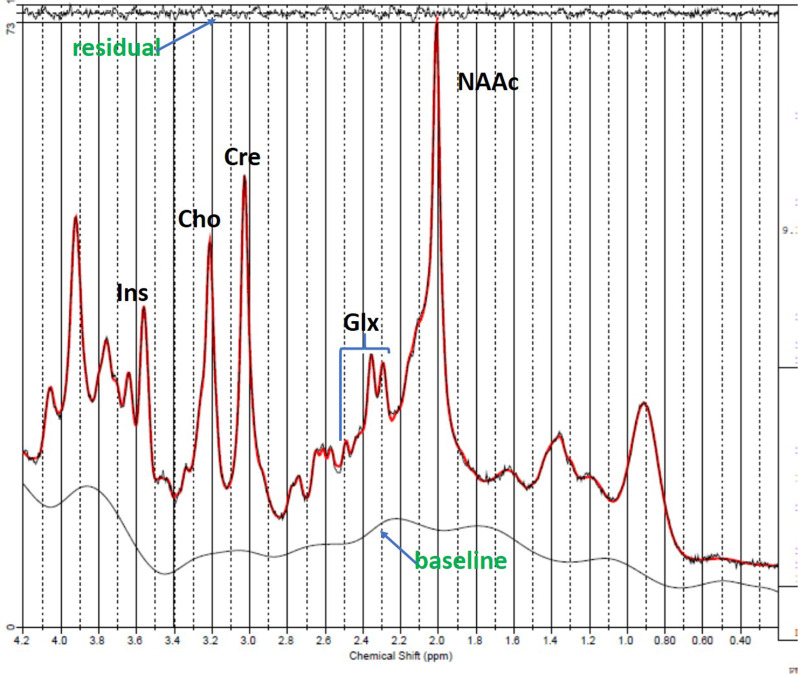
Fitted ^1^H-MRS spectrum (red) acquired from the dorsal anterior cingulate cortex in human. Glx (glutamate plus glutamine), NAAc (N-acetyl-aspartate compounds), Cre (creatine total), Cho (choline compounds), ins (inositol). Used VTE-PR STEAM (TR/TM/TE = 2000/10/6.5, VOI = 6 cm, NEX = 256, 2048 complex points; water scan, NEX = 16) in a Siemens 3T TIM Trio with a 32 channel head coil.

Signals emitted from many protons are converted into a spectrum through a mathematical transformation [38]. Hence, an MRI spectrum has the “signature” frequencies identifying various molecules along the *x*-axis, which are standardized as parts per million (ppm), so that the it is the same at all magnetic field strengths. The amplitudes along the *y*-axis are related to the concentration for each molecule (Fig. 2).

Fitting the area under each of the metabolite peaks and referencing the value to water in the same tissue, provides an estimate of molecular concentration. Though in principle, any molecule containing protons that can resonate will generate a specific frequency, in practice only molecules that are at high enough concentrations and are small and mobile (ie: dissolved in water) will produce detectable signals. However, the larger the volume of interest, the longer the acquisition time and stronger magnetic field, the greater the resonance signals will be. Hence, ^1^H-MRS voxels of interest are usually in centimeters while MRI, which uses the resonance from protons attached to water, has a spatial resolution in millimeters.

While in 5 min a whole brain MRI with tens of thousands of voxels is easily acquired, with ^1^H-MRS, one voxel of a few cubic centimeters is typically collected within that same time. At field strengths of 1.5 Tesla (T), the typical molecules detectable in brain are N-acetylaspartate, creatine and choline. At 3 and 4T, glutamate (Glu) can also be detected. At 7T, glutamine, GABA and glutathione are additionally resolved. In schizophrenia there have been over 130 studies published examining group differences in glutamate, its metabolite glutamine (Gln) and combinations of Glu and Gln (Glx) in different brain regions across various stages of the illness. The great majority of the studies reviewed below were implemented at 3T. We explicitly mention field strength for the minority of investigations at 7T.

### Cross-sectional case-control ^1^H-MRS studies

The two initial meta-analyses examining Glu, Gln, and Glx had somewhat opposite results. Marsman et al. included 647 patients with schizophrenia and 608 HV, found reduced Glu in medial frontal cortex but increased Gln in the same region [39]. Merritt et al, included 1686 schizophrenia patients and 1451 HV, reported increased Glu and Glx in the basal ganglia (including the caudate, putamen and pallidum), elevated Glx in the medial temporal lobe and increased Gln in the thalamus [40]. More recently, two studies have further quantitatively summarized the ^1^H-MRS glutamate literature, a new meta-analysis (Nakahara et al. [41]) and a mega-analysis (Merritt et al. [42]).

Nakahara et al. included subjects in the full schizophrenia spectrum: at high risk for psychosis (HR), patients with first-episode psychosis (FEP), or patients with established schizophrenia (medicated and unmedicated), as well as treatment-resistant schizophrenia (TRS) and HV [41]. One hundred and thirty four studies, involving 7993 subjects with schizophrenia-spectrum disorders and 8744 HV were included and effect sizes (*g*) were examined in 14 regions of interest (ROIs). Most studies presented data for Glx (n=84), fewer for Glu (*n* = 68) and only 11 reported for Gln. In the whole group, Glx levels in the basal ganglia were elevated (*g* = 0.32). There were no Glu differences between the whole clinical group (all schizophrenia subjects, excluding HR samples) vs HV in any ROI, but subgroup analyses revealed various other effects that are discussed below. There were no Gln group differences in any ROI even when focused on only the studies at 7T (seven studies), which had the greatest resolution to separate Glu from Gln. In the HR group, Glu levels were increased in the basal ganglia (g=0.89) whereas in the thalamus Glx and Glu were reduced (*g* = −0.28 and −0.2, respectively).

Another recent meta-analysis of HR ^1^H-MRS studies [43], reported results somewhat contrary to Nakahara et al. [41]. There were no Glx differences in basal ganglia (but did not analyze Glu). However, in the thalamus Glx was increased (*g* = 0.36) but only in “genetic” HR groups (the “clinical” HR sample tended to have increased Glx, though non-significantly). These findings suggest that HR samples are unlikely to be homogeneous in terms of glutamatergic metabolism and further attention to the distinction between genetic and clinical HR is warranted.

Nakahara et al. [41] also found that combined FEP and unmedicated schizophrenia patients had elevated Glx levels in the hippocampus (*g* = 0.47) and dorsolateral prefrontal cortex (*g* = 0.25), but not in the dorsal anterior cingulate cortex (dACC; consistent with a recent large study in antipsychotic-naïve FEP (*n* = 70) that reported negative findings in the dACC [44]). Patients with TRS had increased Glx (*g* = 0.7) and Glu (*g* = 0.63) in the mid cingulate cortx (MCC) [41]. However, MCC Glu was reduced (*g* = −0.17) in the schizophrenia group that did not include the TRS patients. These frontal Glu differences were rather selective to MCC and not detected in the ACC or the larger medial frontal cortex (MFC; which includes both ACC and MCC). Thus, while patients with schizophrenia tend to have reduced Glu in the MCC, a subgroup of TRS may have higher levels in this region. This is somewhat consistent with a more recent study in TRS (*n* = 41) that reported increased Glu in the ACC [45]. In summary, the Nakahara et al. (2021) meta-analysis suggests that through every illness stage, even before onset of psychosis, Glx levels in the basal ganglia are increased, though the group differences are reduced in patients with antipsychotic treatment, consistent with longitudinal studies (see below).

To further examine the effects of more specific clinical characteristics, like symptoms and antipsychotic medications, Merrit et al. (2021) compiled and analyzed subject level data from 1251 patients with schizophrenia and 1197 HV in two ROIs [42]. In broadly defined MFC, Glu, and Glx levels were lower in schizophrenia. There were no differences in medial temporal lobe (MTL; which included the hippocampus). This is somewhat consistent with the Nakahara et al. [41] schizophrenia subgroup findings in MCC (reduced Glu in whole schizophrenia subgroups). Furthermore, antipsychotic dose was negatively associated with MFC Glu (estimate, 0.10 reduction per 100mg chlorpromazine/equivalents). Only one ^1^H-MRS metric was associated with illness severity: Glx/Creatine in the MTL, was mildly positively correlated with total symptom severity (estimate, 0.06) and this was mostly accounted by an effect of negative symptoms. These results suggest that frontal Glu levels may be associated with greater illness severity. However, Glu may be reduced through effective antipsychotic treatment to levels below those observed in HV.

### Schizophrenia vs bipolar psychosis

Broadening the perspective on glutamatergic dysregulation in other psychiatric disorders may further understanding of the NMDA-R hypofunction model of schizophrenia. Because about 50% of bipolar-I (BP-I) disorder individuals experience psychotic symptoms often indistinguishable from schizophrenia, this is a particularly important group to examine.

Few studies have directly compared glutamatergic indices in schizophrenia and BP-I subjects. Three studies failed to detect any differences in chronically-treated schizophrenia and BP groups [46–48]. More recently, our group measured whole brain Glx (over 8000 voxels) in FEP [49]. Forty-eight schizophrenia, 21 bipolar-I subjects with history of psychosis and 51 HV were studied. Schizophrenia subjects had higher Glx in the right middle cingulate gyrus than BP-I subjects. Healthy volunteers had intermediate Glx values.

However, the elevated Glx in schizophrenia was accounted for mainly by the antipsychotic-naïve subjects (39% of schizophrenia and 40% BP-I patients were antipsychotic-naïve). Glx was not related to symptomatology. The advantage of this study is the use of voxel-wise analyses using family-wise correction akin to the rest of brain imaging. Hence, early in the course of psychotic illness, schizophrenia and bipolar disorders may fundamentally differ in their glutamatergic metabolism in particular cortical areas but further studies with larger samples are needed.

### Longitudinal ^1^H-MRS studies in schizophrenia

Not surprisingly, there are fewer longitudinal studies examining the effects of change in state (severity of psychosis), of disease progression and of antipsychotic medications (both beneficial and deleterious) on glutamatergic measures. Kubota et al. [50], recently implemented a meta-analysis of 32 studies (*n* = 773 at follow-up). They reported that frontal Glx was decreased (effect size = −0.35) after antipsychotic treatment in schizophrenia patients. There were no associations with duration of treatment, or symptom changes. Below, we discuss a few particularly informative individual studies including some more recent ones not included in the meta-analysis.

De la Fuente-Sandoval et al. [51] studied 24 patients with first FEP and HV [51]. This is a particularly informative study because patients were antipsychotic-naïve and with no history of substance use. FEP had higher levels of Glu in the associative striatum and the cerebellum at baseline (Glx was also increased in the striatum at baseline, consistent with the Nakamura et al., meta-analysis, 2022). After 4 weeks of treatment with risperidone, Glu was decreased in the associative striatum, with no significant change in the cerebellum.

A follow-up study by the same group in 48 antipsychotic-naïve FEP [52], found that baseline striatal Glu was higher in the non-responders than responders both pre- and post-treatment, suggesting that Glu modulation (reduction of increased levels) may be linked to the dopamine blocking effects of antipsychotics in the striatum. Because increased dopamine synthesis has been documented in schizophrenia in the associative striatum independent of antipsychotic exposure [53], it is tempting to expect a positive relationship of Glu and dopamine synthesis in this region. However, the only study to date that (to the best of our knowledge) has examined both Glu and dopamine in the striatum in schizophrenia (of treated patients) found a negative correlation [54]. Conversely, one study in healthy volunteers reported a positive correlation of striatal dopamine and Glu in HV [55].

In a multicenter study, Egerton et al. examined 71 minimally treated FEP before and after 4 weeks of therapy with amisulpride [56]. There were no differences in baseline Glu/Cr between patients and controls. However, higher Glu/Creatine (Cr) in the ACC were associated with more severe symptoms at baseline and a lower likelihood of responding to treatment. There were reductions in Glu/Cr in both the dACC and thalamus with treatment. However, Glu/Cr changes were not related to symptom improvement. This study had several strengths like having the largest longitudinal sample to date and having standardization of treatment. However, Cr has been found to be reduced in schizophrenia in MFC [42] and to be affected by antipsychotic treatment [49]. Hence, referencing Glu to Cr complicates the interpretation of findings.

Bojesen et al. [57] studied 39 antipsychotic-naïve FEP patients 36 HV with repeated Glu/Cr measurements of ACC and thalamus [57]. Before treatment, thalamic Glu/Cr was higher in the whole group of patients but levels normalized after treatment. However, ACC Glu/Cr was lower at all assessments and unaffected by treatment. FEP patients with highest thalamic Glu/Cr at baseline were the least likely to respond to treatment. Early and late assessments is a clear strength of this study. The results suggests that the effect of antipsychotics on Glu may vary by region. Still, referencing to Cr limits the interpretation.

Another in minimally-treated FEP (*n* = 21) found reduced baseline Glu in dACC but no changes after six months of treatment [58], consistent with Bojesen et al. [57]. Strengths of the study included longer follow-up to assess disease progression and spectral higher resolution (at 7T). More recently, Li et al. (2022) also examined MFC Glu in a longitudinal study of antipsychotic-naïve FEP (*N* = 32) [59]. There were no baseline differences with HV and no changes after 8 weeks of antipsychotic treatment. However, they used a 27 cc MEGAPRESS voxel optimized for GABA measurement and probably less sensitive to Glu changes.

In the largest longitudinal follow-up study focused on the hippocampus, Kraguljac et al. [60] examined 61 unmedicated schizophrenia patients before and after 6 weeks of treatment with risperidone [60]. They found increased (Glx) at baseline but no effect of antipsychotic medication. Also there was no relationship to symptom changes.

One study has longitudinally examined the effects of clozapine on Glu. In a TRS population (*N* = 25), McQueen et al, found that 12 weeks of clozapine therapy was associated with a reduction in Glu in the caudate but not in the ACC [61]. Percentage reduction in caudate Glu was positively correlated with improvement in symptoms.

In summary, though a meta-analyses of 6 longitudinal studies supports frontal Glx reductions in the context of AP treatment, three new studies in FEP have failed to document frontal Glu reductions. However, one new study in TRS did find ACC Glu reduction with clozapine treatment. Clearly, more studies are necessary to confidently ascertain any glutamate modulating effects of antipsychotic medication exposure and whether these may be related to their therapeutic or deleterious neuro-motoric effects.

### Functional ^1^H-MRS

Because relatively fast (in minutes) glutamatergic changes have been detected in HV with ^1^H-fMRS in the context of cognitive challenges [62], three groups have applied this approach to schizophrenia populations, all focused on the dACC. Taylor et al. used the Stroop task at 7T [63]. Healthy controls (but not schizophrenia or major depression subjects) demonstrated a significant Glu increase (3.2%). Similarly, Jelen et al. [62], reported increased Glu/Cr during an N back task in healthy controls but not in schizophrenia or BP-II subjects. However, Chiappelli et al. (2017) detected increments of Glu in schizophrenia patients compared to HV during a painful stimulus [64]. Hence, functional ^1^H-MRS is very early in its application to schizophrenia research but may hold great promise.

In summary, most of the neuroimaging literature investigating glutamate in schizophrenia has involved single-voxel ^1^H-MRS case-control studies. Increased glutamate in basal ganglia structures, across most stages of the illness (even before antipsychotic medication) has been documented, as well as medial frontal glutamate reductions in chronically-ill populations. The more limited longitudinal literature suggests that in both structures antipsychotic medications appear to foster glutamate decrements. These findings only in part support a NMDA-R hypofunction model of schizophrenia.

## Cerebrospinal fluid and peripheral blood findings

The NMDA-R hypofunction hypothesis was reinforced by an early hallmark study showing lowered glutamate levels in CSF among a small population with schizophrenia as compared with HV [65]. These findings were however never replicated. Conversely, a later study did not demonstrate any difference in autopsied whole brain and CSF levels of glutamate among schizophrenia patients compared to HV [66].

Another study examining in vivo Glutamate levels in CSF did repeat these findings, but they also showed no concentration difference between patients concurrently treated with haloperidol as well as patients without any ongoing antipsychotic treatment [67]. In summary, these findings are indicative that the effect of glutamate on the pathogenesis of schizophrenia is related to other mechanisms than through its concentration in CSF.

More recently anti-NMDA-R encephalitis has been recognized as an autoimmune disorder sometimes presenting with psychotic symptoms. It is caused by autoantibodies against an extracellular domain of the NMDA-R. It presents clinically with aggression, catatonia, seizures, forgetfulness and frank psychosis. Since then, several case-control studies have measured NMDA-R antibodies in plasma.

One initial study reported that 6.5% of with FEP were shown to be NMDA-R-antibody positive [68]. In a similar study, Lennox et al. enrolled 228 FEP and 105 HV. Seven (3%) patients had NMDA-R antibodies compared with no controls [69]. However, there have been negative studies that used qualitative methods such as immunofluorescence or immunohistochemistry [70, 71].

Pollak et al. [72] reported a meta-analysis of schizophrenia patients (*n* = 1441). Of these, 21 (1.46%) patients were positive for antibodies of the IgG subclass. Furthermore, only for IgG antibodies were prevalence rates significantly greater in FEP than controls [72]. Another study has examined the relationship between NMDA-R antibody levels and clinical features in FEP antipsychotic-naïve patients in which 110 FEP and 50 healthy controls had serum NMDA-R antibody levels measured via enzyme-linked immunoassay (ELISA). As a group, FEP had elevated levels of anti-NMDA-R antibody compared to controls (9.2 ± 3.5 vs. 7.3 ± 2.9 ng/ml).

In FEP, antibodies were positively correlated with both positive and negative symptoms and inversely correlated cognitive function. The group differences were small and no analyses to assign subjects by group based on thresh-hold antibody levels were presented [73]. Hence, a small minority of FEP patients are anti-NMDA-R antibody positive and some persons without psychosis will also test positive. This literature supports the validity of the NMDA-R hypofunction model of schizophrenia but not the generalizability of this specific autoimmune mechanism. It remains to be established whether this subset of patients differs from antibody-negative patients in terms of underlying patho-physiology and response to standard or immunomodulatory treatments.

## Postmortem studies

Altered glutamatergic neural circuitry has been observed in post-mortem brain tissue of schizophrenia patients [30]. This includes structural alterations in glutamatergic neurons, as well as changes in mRNA and protein expression of molecules involved in glutamate signaling (glutamate receptors, glutamate transporters, glutamate synthesizing enzymes, glutamate receptor co-agonists), although some inconsistencies have also been reported [30]. (Dorph-Petersen et al. [74], McCullumsmith et al. [75] and McCullumsmith et al. [76] for reviews on technical issues in psychiatric post-mortem studies [74–76]).

In a comprehensive narrative review Hu et al. [30], summarized studies of glutamate neuron morphology, of the synthesizing and metabolizing enzymes for glutamate and its co-agonists and of the expression of glutamate transporters and receptors. They reported clear evidence in several regions, from multiple labs of reduced dendrite length and complexity and of lower levels of synaptophysin, a marker of axon boutons. Additionally, reduced pyramidal somal volume has been found predominantly in layer III of dorsolateral prefrontal cortex (DLPFC) and auditory cortex. Hence, the dendrites, axons and cell bodies of cortical glutamatergic neurons are clearly reduced in size in schizophrenia.

Regarding the glutamate metabolic pathways, they also found some limited evidence of altered expression of the excitatory amino acid transporter-2 (synaptic re-uptake), glutamine synthetase (glial change of glutamate to glutamine), and glutaminase (presynaptic change from glutamine to glutamate prior to vesicular re-packaging). Finally, they reported no consistent alterations in glutamate receptors. However, a more recent meta-analysis found otherwise.

Catts et al., 2017 implemented a quantitative review of mRNA or protein levels of the obligatory NR1 subunit for the NMDA-R in prefrontal cortex from schizophrenia vs HV contrasts [77]. In the five studies (schizophrenia = 94, HV = 82) of mRNA expression there was a significant reduction (effect size = −0.64) in schizophrenia. For the five studies examining protein expression (schizophrenia = 95, HV = 95) there was also a significant reduction (effect size = −0.44). The other studies of various NMDA-R subunits and different regions of interest studies did not permit additional meta-analyses. A qualitative review of studies of the NR2 (A, B, and D) and NR3A subunits suggested no consistent group differences (however, power analyses suggested that a sample >200 per group would be needed for detection of smaller differences). Regarding the NR2C subunit in prefrontal cortex, though meta-analysis was not performed, mRNA expression was significantly decreased in the three published studies [78–80].

Because excitation and inhibition are intimately related in the brain, Dienel et al. re-analyzed data from six previously published studies and focused on indexing glutamatergic and GABAergic neurotransmission in the DLPFC of 57 schizophrenia and HV matched pairs [81]. They generated overall glutamate and GABA measures by combining several key transcripts involved in synthesis (glutamine synthetase, and glutamic acid decarboxylase), vesicular transport (glutamate transporter 1, GABA transporter), synaptic reuptake (excitatory amino acid transporter 2 and GABA reuptake transporter 1) and critical postsynaptic receptors (GRIN_1_, GRIA_2_, and GABRG_2_). The composite glutamate measure was higher in schizophrenia in total gray matter but reduced in samples restricted to coritcal layer III. The index of GABA neurotransmission did not differ between groups in total gray matter but was lower in schizophrenia in layer III. Hence, this study highlights the inherent limitation of examining ROIs involving several cubic centimeters in ^1^H-MRS studies, since the level of anatomical resolution examined (whole cortex vs layer III) can yield opposite glutamatergic abnormalities.

Wang et al. examined agonist-induced mGluR_5_ signaling in DLPFC of 17 schizophrenia and HV matched pairs [82]. There was significant attenuation of mGluR5 signaling in schizophrenia which was related to NMDA-R hypofunction. These findings were not seen in tissue from monkeys chronically exposed to antipsychotics.

More recently Yonezawa et al. reported results of a systematic narrative review of 39 studies of AMPA-R expression in schizophrenia [83]. There was data examining five regions: frontal cortex, medial temporal lobe (MTL), cingulate cortex, thalamus and striatum. Only in the MTL were there consistent findings (6 of 8 studies) with reduced AMPA receptor binding involving GluA1 and GluA2 in schizophrenia.

Post-mortem studies have a level of structural and molecular resolution orders of magnitude greater than ^1^H-MRS studies. However, they also have limitations. Samples sizes are in the few 10s and regions of interest are limited one or two. Also, samples are typically collected from chronically ill subjects exposed to antipsychotic and other medications for years. Longitudinal studies are of course not available. A few studies have controls of non-psychotic patients chronically exposed to antipsychotics (e.g.,: Huntington’s disease). Some studies present data on the effects of chronic antipsychotic exposure (weeks or months) in rats or monkeys to address this confound. It is possible that some of the dendritic tree retractions may be secondary to antipsychotic medication [84]. Still, post-mortem studies provide a level of molecular resolution that can mechanistically bridge neuro-imaging and genetic data.

In summary, the post-mortem literature reports mRNA and protein levels reductions of the obligatory NR1 subunit for the NMDA-R in prefrontal cortex. The data is not nearly as consistent for AMPA-R. Additionally, the dendrites, axons and cell bodies of cortical glutamatergic neurons are clearly reduced in size in this same region. These findings are consistent with the NMDA-R hypofunction model.

## Genetic studies

Schizophrenia is known to have high heritability (60–90%; [85, 86]) so ascertainment of specific risk genes is critical to understanding its etiology and pathophysiology. Three general approaches have been predominantly employed to detect specific genetic risk factors in schizophrenia studies: GWAS for common variants (each conferring very low risk); whole exome examination of ultra-rare coding variants (potentially conferring substantial risk); and rare CNVs (also with low risk; see O’Donovan et al. [87] and Michaelson [88], for reviews on technical issues in psychiatric genetics [87, 88]).

In the largest GWAS of 76,755 schizophrenia patients and 243,649 control individuals, Trubetskoy et al. (Psychiatric Genetics Consortium (PGC) 2022), found common variant associations conferring risk for schizophrenia at 287 loci [89]. These occurred mainly in genes that are expressed in synapses of both excitatory and inhibitory neurons.

Several risk genes encode voltage-gated calcium and chloride channels known to modulate glutamate receptor activity (CACNA1C and CLCN3), metabotropic receptors (glutamate (GRM1) and GABA (GABBR2)), and the NMDA-R subunit (GRIN2A).

Singh et al. used whole exome examination of rare variants on the largest sample to date [90]. In 24,248 schizophrenia patients and 97,322 controls they detected group differences in 10 different genes. Of these, three were clearly related to glutamatergic function: in the NMDA-R subunit GRIN2A and the receptor subunit GRIA3.

In the largest CNVs study to date (21,094 schizophrenia patients and 20,227 controls), Marshall et al. found eight loci, with deletions that conferred increased risk of schizophrenia [91]. These included various synaptic cell adhesion and scaffolding proteins as well as variants involving glutamatergic ionotropic receptors (GRID_1_, GRID_2_, GRIN_1_, GRIA_4_).

Hence the best powered studies to date with different approaches, have consistently detected several genes directly involved in glutamatergic neurotransmission. In contrast, none of these studies identified any risk genes involved in dopaminergic neurotransmission; in the previous PGC report only 1 out of 108 significant loci involved a dopamine D_2_ receptor gene [92]. Several other risk loci are involved in multiple synaptic functions and likely affect glutamatergic function indirectly. Still, all these glutamatergic related factors account for a very small proportion of the overall heritability in schizophrenia and do not support a major role for genes known to impair NMDA-R function.

## Pharmacological studies

The NMDA-R hypofunction model of schizophrenia has supported the development various mechanistic strategies and testing of compounds usually targeting negative and cognitive deficits but also positive symptoms. A variety of drugs have directly targeted the NMDA-R glycine binding site as co-agonists (Glycine, D-serine, D-cycloserine, or D-alanine).

Another strategy relies on inhibition of the glycine transporter (sarcosine, bitopertin) or of D-amino acid oxidase (DAAO), the enzyme that metabolizes D-serine (sodium benzoate). A different indirect approach involves allosteric modulation of AMPA-R facilitating magnesium removal from NMDA-R and increasing their availability for glutamate stimulation (CX516 ampakine, piracetam, diazoxide). Because NMDA-R hypofunction (for example in GABAergic interneurons) has presumed down-stream effects (dis-inhibition of pyramidal cells) with increased glutamatergic neurotransmission and possible neurotoxicity, low affinity NMDA-R antagonism has been an alternative approach (memantine, riluzole). Also targeting non-NMDA-R with positive allosteric modulation (PAM) of MGlu2/3 (polaglumetad, AZD8529) and of mGlu_5_ receptors (VU040955) has been implemented. Finally, other broader options have involved reduction of presumed glutamatergic excitotoxicity (minocycline, acamprosate, lamotrigine). Below, we briefly review the clinical trial literature.

Early small trials (some unblinded) of glycine and D-serine were encouraging [93–98]. One meta-analysis of only randomized controlled has been conducted [99]. It included mainly males (75%) with persistent negative symptoms. The studies involved add-ons to ongoing antipsychotic therapy. There were no benefits for D-cycloserine (*n* = 119, 5 RCTs). However, for the combined glycine or D-serine studies (*n* = 132, 7 RCTs), there was mild improvement in negative symptoms for the active treatments.

The largest multi-center study to date (*N* = 157, 16 weeks of treatment; not included in the meta-analysis) comparing glycine, D-cycloserine and placebo for cognitive and negative symptoms and found no effect [100]. More recently, in a group at high-risk for schizophrenia, D-serine was beneficial for negative symptoms [101].

Several initial trials of sarcosine were positive [102–105], but a more recent trial (*N* = 63, 12 weeks) showed no improvement in positive and negative syndrome scale or cognitive function when used alone [106]. Bitopertin, another glycine transporter inhibitor had some early encouraging effects [107], but a subsequent much larger study (*N* = 627, 24 weeks) failed to detect an advantage [108]. Smaller studies with sodium benzoate have had some positive results for negative symptoms and cognitive deficits [109, 110], but further confirmation is necessary.

An initial study of the ampakine CX516, documented some cognitive improvement [111]; however, a larger trial (*N* = 105, 4 weeks) showed no improvement in cognition [112]. Small studies with piracetam and diazoxide, other AMPA-R modulators, have showed beneficial effect for positive symptoms [113, 114] but further support with larger studies is missing.

Recently a meta-analysis quantified the effects of more broadly defined positive glutamate modulators of NMDA (benzoate, D-cycloserine, D-serine, glycine, and Org25935) and AMPA (CX516 and minocycline) receptors targeting cognitive deficits in schizophrenia [115]. There were no differences from placebo in any of the cognitive measures.

The NMDA-R blocker memantine had some initial encouraging results [116–119] However, the largest study (N=138, 8 weeks), had negative results [120]. One small trial with riluzole had positive effects [121], but follow-up studies are necessary.

Probably the most robust efforts to test glutamatergic drugs in schizophrenia have involved the development of PAMs of metabotropic receptors. Pomaglumetad is a presynaptic mGluR 2/3 agonist that reduces glutamate release only when synaptic concentrations are high. In an initial large study (*N* = 196, 4 weeks) in exacerbated schizophrenia, pomaglumetad showed antipsychotic efficacy compared to placebo and olanzapine [122]. However, a larger follow-up study (*N* = 667, 4 weeks) was negative [123]. Another mGlu_2_ PAM (AZD8529), also failed to show efficacy [124].

Still, a re-analysis of the large pomaglumetad trial suggested positive antipsychotic effects in schizophrenia patients very early in the illness who were previously unexposed to antipsychotic drugs [125]. In addition, a more recent trial of HV showed greater reduction of ketamine-induced positive symptoms with higher (320 mg/d), compared to lower (80 mg/d), pomaglumetad dosing [126]. This suggests that pomaglumetad dosing may have been inadequate in prior clinical trials. Also, consistent with some of the ^1^H-MRS literature reviewed above, there is the possibility that some PAM mRGlu2 agents may be effective for a subgroup of patients who have greater increases in glutamate in certain brain areas like the striatum.

The tetracycline antibiotic minocycline likely affects glutamatergic metabolism in multiple ways, presumably reducing the likelihood of excitotoxicity. Small trials have been add-ons to antipsychotic agents targeting mainly cognition and negative symptoms and have been mixed [127–130]. The largest of these, a multi-center RCT (*N* = 144, 52 weeks) reported improved negative symptoms [131]. However, the effects were mainly accounted by one of the sites. One small trial of acamprosate had negative results on psychotic symptoms [132].

Lamotrigine, stabilizes pre-synaptic sodium-channels by inhibiting glutamate release. Five add-on RCTs have been done (*n* = 537). However, a meta-analysis could only include data for 70 schizophrenia subjects. In this limited sample there was a small global improvement on symptoms of questionable clinical significance [133].

In summary, the large clinical research effort of glutamate modulating agents for schizophrenia has so far resulted in no available treatments. Two main strategies informed by the NMDA-R hypofunction model have been systematically pursued: potentiation of the glycine NMDA-R site in add-on RCT targeting persistent negative and cognitive deficits (e.g., glycine, D-serine, sarcosine); and downregulation of presynaptic glutamate release with PAMs of metabotropic receptors as a full antipsychotic alternative to dopamine D_2_ blockade (e.g., pomaglumetad). Failure of these glutamatergic strategies is consistent with the lack of efficacy of various other non-dopaminergic approaches including adjunctive pharmacotherapy with anticholinesterase inhibitors, antidepressants, anti-oxidants, mood stabilizers, anti-inflammatory agents, hormone replacements or stimulants [134]. Clearly, further elucidation of the pathophysiology of schizophrenia is critical to the development of novel therapeutic strategies that go beyond dopamine D_2_ blockade.

## Conclusions and future directions

The NMDA-R hypofunction model of schizophrenia started with the clinical observation of the precipitation of psychotic symptoms in individuals having been exposed to PCP through anesthesia or by recreational use [135]. These descriptions were expanded to healthy volunteers exposed to controlled low doses of ketamine whom consistently experienced mild psychosis but also negative and cognitive domain symptoms reminiscent of the full clinical picture of schizophrenia [18]. In various models using awake rodents, it was ascertained that systemic pharmacological acute NMDA-R blockade resulted in a paradoxical increase in extracellular frontal glutamate as well as of dopamine. Similar increase in prefrontal glutamate has been documented with acute systemic ketamine in healthy volunteers with ^1^H-MRS. Furthermore, in rodents exposed chronically to low dose PCP, reductions in frontal dendritic tree density have been observed.

In post-mortem ultrastructural studies in schizophrenia, a broad reduction in dendritic complexity and somal volume of pyramidal cells has been repeatedly described, without gliosis or neuronal death. In other post-mortem literature, moderate prefrontal reductions in the obligatory GluN_1_ subunit of the NMDA-R has been reported. The vast ^1^H-MRS literature in schizophrenia has documented small increases in glutamate (or Glx) concentrations in basal ganglia (mainly the caudate nucleus) very early in the illness, before antipsychotic treatment. These increases persist later in the course of the illness, but appear to be moderated by treatment. In other brain regions, findings are more complex with glutamate reduction in medial cingulate cortex apparently related to antipsychotic exposure. Consistently, longitudinal studies tend to find frontal glutamate reductions following treatment. However, the literature does not support associations between symptom severity and levels or changes in ^1^H-MRS measured glutamate.

The more recent genetic literature with samples in the tens of thousands, has reliably detected very small risk effects for common variants involving several glutamate-related genes. Alternative approaches examining rare-variants have also documented the association with glutamatergic genes. However, the risk conferred in the overall prevalence of schizophrenia by both the common and rare variants is very small. Still, the current data supports the notion that the genetic liability of schizophrenia more directly involves the glutamatergic than the dopaminergic system. The RCT literature has followed two main tracks, directly informed by the NMDA-R hypo model: agonism at the glycine site; and pre-synaptic modulation of glutamatergic release. The former approach has involved a variety of agents examined in many small add-on studies, focusing mainly on negative symptoms and cognitive deficits. The later approach has involved few larger studies of specific PAMs targeting acute psychosis as a single agent. Unfortunately, both approaches have failed so far.

What is the meaning of these findings? There is little doubt that brain glutamatergic abnormalities are present in schizophrenia and that some of these are related to the etiology of the illness. Whether NMDA-R hypofunction as a specific mechanism accounts for any important component of the illness is still not evident. The genetic literature directly supports a non-specific etiological role for glutamatergic dysfunction. However, although highly heritable, schizophrenia is a polygenic disease with likely thousands of variants contributing to the risk. The few genetic loci directly underlying glutamate neurotransmission identified to date, have a minimal contribution to the overall disease risk. ^1^H-MRS data clearly shows increased glutamate in striatal areas, similar regions to where dopamine release is increased per PET data. This is not accounted by antipsychotic exposure and the two neurotransmitters systems are known to be associated in the striatum.

Although it is tempting to assume that glutamate and dopamine striatal increases in schizophrenia are related, the very few data available does not support this claim (a model of dopamine and glutamate interaction potentially underlying treatment-resistant schizophrenia has recently been proposed [136]). Increased and reduced Glu or Glx in other regions and different stages of the illness are harder to confirm and interpret.

However, within the NMDA-R hypofunction model it is plausible to expect increases in glutamate in some areas whose glutamatergic cell bodies have been dis-inhibited due to a primary NMDA-R hypofunction in GABA-ergic interneurons, as originally postulated by Olney and Farber [8]. Later in the illness, some of the dendritic trees impacted by this dis-inhibited glutamatergic input, may plastically retract to blunt the risk of more serious excitotoxicity, and present with overall glutamate reductions in ^1^H-MRS. This would be relatively consistent with the longitudinal MRI literature that supports progressive widespread cortical thinning in schizophrenia [137], and with the post-mortem ultrastructural data showing neuropil reductions as well as NMDA-R decrements. The clear failure of the current RCT literature does not support the overall NMDA-R hypofunction model as critical to the pathophysiology of schizophrenia. Still, it is important to discuss some of the major limitations of these past studies in order to clarify future paths for research.

The neuroimaging literature is basically restricted to ^1^H-MRS studies. These suffer from limited spectral (Glu, Glx, or Gln) and spatial resolution (2–12 cm3 single voxels) as well as very sparse spatial coverage (1 to 5 voxels). The human cortex is 2–3 mm thick, so even a relatively small 1–2 cm3 voxel of folded cortex will include a large proportion of white matter and CSF. This large partial volume effect can only be addressed statistically, and it likely affects the reliability of spectral measurements. Even at 7 T, voxels tend to be in the several cm3 (the largest 7T psychosis study to date collected 5 voxels that ranged between 8 and 12 cm3 each; [138]).

If, as one post-mortem studies suggest [81], glutamate concentrations in different cortical layers can vary in opposite direction in schizophrenia, the restricted spatial resolution is an even more critical limitation (even the available 1 mm3 resolution of most other MRI techniques cannot yet address this hurdle). The problem of spectral resolution and the validity of static whole tissue metabolite measurements has been partially addressed by increasing field strength, using spectral editing techniques that further resolve a peak of interest and functional ^1^H-MRS with some limited improvement.

Another approach to more directly measure glutamate neurotransmission has been the use of 13C-MRS. This technique can measure the Glu/Gln cycling dynamically. Because most of the brain’s Gln is generated from Glu re-uptake from the synapse, this cycling is a more valid measure of neurotransmission than static metrics of Glu or Gln. Consistent with the NMDA hypofunction model, in healthy controls and depressed subjects, systemic ketamine induced increased Glu/Gln cycling in the prefrontal cortex [139]. However, the signal-to-noise from 13C-MRS is such that human studies generally require very large regions of interest and long acquisitions (over half of the frontal lobe for 120 min [139], contrasted with 5 to 10 min for single voxel ^1^H-MRS and 17 min for EPSI). The issue of spatial coverage is critical for an illness like schizophrenia, where essentially very small structural effects over the whole brain, have been described [140]. Furthermore, it is far from established which regions are fundamentally affected in the illness. Hence, it would be very irregular for an fMRI, diffusion tensor imaging or structural MRI study to examine only 1 or 3 regions of interest in a study of schizophrenia, following the claim that these regions are “known” to be involved in the illness. However, this is precisely the state of the current ^1^H-MRS literature where the overwhelming majority of studies use the single voxel approach. Whole brain approaches, like EPSI [49] or GluCEST [141], improve coverage to over 2/3’s of the brain. However, important frontal and basal regions are not well covered with these approaches.

Eventually, the development of various safe ionotropic and metabotropic-binding radio-nuclides should allow the next generation of PET neuroimaging to proceed and a more thorough testing of the NMDA-R hypofunction model of schizophrenia.

These would involve a progression of more demanding/informative studies as has been done measuring dopaminergic function: initially case-control studies of treated schizophrenia, followed by antipsychotic-naïve schizophrenia, bipolar-I with psychosis and high-risk psychosis (measuring the strength and specificity of NMDA hypofunction in schizophrenia); then longitudinal studies in schizophrenia before and after standard antipsychotics (measuring the relationship between change in psychosis and NMDA hypofunction); finally, very expensive and large longitudinal cohort studies of high-risk subjects before and after transition into psychosis (measuring the degree that hypofunction predicts conversion to psychosis). Because of the invasiveness and cost of PET, even if a good ligand is developed, it would take many years to implement such a program of research.

Still, even these hypothetical neuroimaging studies would remain descriptive and they would have to be complemented by new experimental animal models as well as post-mortem studies to discover novel potentially drug-able molecular targets. Brain transcriptomics, a quantitative genome-wide molecular approach that examines post-mortem gene-expression, has documented synaptic down-regulation in schizophrenia and BP-I [142]. These synaptic gene-expression reductions were related to polygenic (SNP-based) disorders differences, suggesting a significant causal genetic component. Larger post-mortem transcriptomic studies maybe be able to further specify the degree to which synaptic down-regulation may be related to glutamatergic dysfunction.

The post-mortem approaches complement the neuroimaging methods: they provide molecular and ultra-structural resolution as well as much more solid mechanistic interpretations. However, they are generally an end-point static view of a patho-physiological process that may have started in-utero, progressed during early development and adolescence and is affected by multiple con-founding factors which are the norm (e.g., antipsychotic medication, substance use, metabolic syndrome). The effect of antipsychotic drugs can be particularly problematic. For example, chronic exposure (17–27 months) in rhesus monkeys to clinically-relevant dosages of olanzapine and haloperidol resulted in an 8% volume reduction in gray and white matter across most brain regions [143]. This appeared to be accounted by a 14.2% lower glial cell number and a compensatory 10% increase in neuronal density, though not neuronal count [84]. Some molecular glutamatergic abnormalities described above are unlikely to be secondary to antipsychotic exposure [81]. However, chronic antipsychotic exposure may account for some of the dendritic tree reduction and progressive cortical thinning repeatedly described in schizophrenia. Modeling tissue function without the confound of antipsychotic medication may be possible using induced pluripotent stem cell (iPSC) technology. This approach generates differentiated neuron-like cells that retain the full genetic information of the patient with schizophrenia [144].

Probably the most striking message from the genetic literature is that despite the very robust heritability of schizophrenia, the very small genetic effects in many risk loci, will continue to require larger and larger samples (i.e., tens or hundreds of thousands), to be able to account for a large proportion of variance in the risk for the illness. This poses a huge practical challenge for future studies examining genetic/neuroimaging (^1^H-MRS or PET) relationships that hope to connect the etiological risk of specific genes with brain pathophysiological mechanisms. Also, the complexity of the genetic underpinnings of schizophrenia requires a re-assessment of more traditional animal models of the illness.

Just as candidate-gene approaches to human studies are no longer credible, the development of animal models involving a few genes are unlikely to plausibly represent the genetic architecture of schizophrenia. However, as further knowledge develops regarding the non-coding functions of the majority of DNA, new paradigms will evolve that can be implemented in human samples.

Circular RNAs (circRNAs) are non-coding RNAs, are abundantly expressed in mammalian brain (especially in the prefrontal cortex), many pass through the blood-brain barrier and can be quantified peripherally [145]. Their expression is higher during development as well as in the context of neuronal activity and specifically in synaptic plasticity. Their precise function is unknown but they appear to exert diverse regulatory effects in protein-coding gene expression. Recently, deficits in circ-Homer1 in both the prefrontal cortex and IPSC-derived neuronal cultures were documented from patients with schizophrenia and bipolar disorder [146]. The HOMER1 protein constitutes a major part of the post synaptic density and is intimately involved in glutamatergic neurotransmission. Future studies measuring thousands of circRNAs in blood from psychotic patients in conjunction with brain measures of glutamate with whole brain ^1^H-MRS, may be a new avenue to elucidate the link between genetic-risk and neurobiological expression in glutamatergic and other systems.

The extent of the RCT literature documents the impact that the NMDA-R hypofunction model has had over the last three decades. Unfortunately, this vast research endeavor has not resulted in any new choices for the clinician. Heterogeneity of the illness, not only in terms of phenomenology (persistent primary negative symptoms or acute psychotic exacerbation) or course (early vs late), but also abscense of putative endophenotypes (increased striatal glutamate, reduced mismatch negativity), is a common post-hoc explanation for negative RCT findings.

Interpretation of the negative RCT literature in the context of ^1^H-MRS findings is complex. Because of ^1^H-MRS results supporting glutamate reductions secondary to antipsychotics, agents developed to increase glutamate, like NMDA agonists, would be unlikely to have a therapeutic role (perhaps beyond correcting some side-effects of D_2_ blockers). However, since increased glutamate in striatum has been documented in schizophrenia before D_2_ treatment, some glutamatergic agents for patients that failed D_2_ blockers and have persistently increased glutamate may still have a role [136]. Additionally, negative symptoms and cognitive deficits generally are not improved by D_2_ blockers. Also, frontal glutamate reductions are found in chronically-ill patients. Hence, negative symptoms and cognitive deficits were reasonably targeted with NMDA agonists. Finally, a PAM agent like pomaglumetad which is a presynaptic mGluR 2/3 agonist, could have been expected to reduce glutamate release when synaptic concentrations are high and increase them when they are low, a goal not inconsistent with ^1^H-MRS findings. Finally, though disappointing, the failure of glutamatergic drugs is not an anomaly in schizophrenia pharmacology. Besides dopmaninergic D_2_ blockers for positive symptoms, no other compound has demonstrated efficacy for any component of the illness. These have included a variety of non-glutamatergic strategies examined in RCT’s like: serotonergic modulators, non-steroid anti-inflammatory, estrogen action, lithium, adenosine modulators, azapirones, modafinil, testosterone, oxytocin, pregnenolone, anticholinesterase inhibitors, histamine H_2_ blockers, varenicline, davutenide and benzodiazepines [134].

In summary, genetic, post-mortem and neuro-imaging research support broad glutamatergic abnormalities in the brain of schizophrenia subjects. Some of these abnormalities are consistent with the NMDA-R hypofunction model of schizophrenia and they are not secondary to common clinical confounds. However, the extent to which they represent causal mechanisms as opposed to compensations or consequences of more fundamental brain deficits is not clear. Furthermore, these glutamatergic abnormalities do not correlate with important components of the illness, like positive or negative symptoms or cognitive deficits. Informed by these findings and by the NMDA-R hypofunction model, a variety of pharmacological strategies have been tested and have to date, failed. However, a glutamatergic model still has heuristic value to guide future research in schizophrenia. New tools to jointly examine brain glutamatergic, GABA-ergic and dopaminergic systems in-vivo, early in the illness, may lay the ground for a next generation of clinical trials that go beyond dopamine D_2_blockade.
